# Withaferin A Acts as a Novel Regulator of Liver X Receptor-α in HCC

**DOI:** 10.3389/fonc.2020.628506

**Published:** 2021-01-29

**Authors:** Varsha D. Shiragannavar, Nirmala G. Sannappa Gowda, Divya P. Kumar, Faridoddin Mirshahi, Prasanna K. Santhekadur

**Affiliations:** ^1^ Department of Biochemistry, Center of Excellence in Molecular Biology & Regenerative Medicine, JSS Medical College, JSS Academy of Higher Education and Research, Mysore, India; ^2^ Division of Gastroenterology, Hepatology and Nutrition, Virginia Commonwealth University, Richmond, VA, United States

**Keywords:** Withaferin A, hepatocellular carcinoma, migration, invasion, angiogenesis, proliferation

## Abstract

Withaferin A, a steroidal lactone derived from the *Withania somnifera* plant has been known for its anti-cancerous effects on various types of cancer cells. However, its effect on the hallmarks of cancer such as proliferation, migration, invasion, and angiogenesis is still poorly understood. The antitumor property of Withaferin A and its molecular mechanism of action on hepatocellular carcinoma (HCC) cells is not yet completely established. In this study, we aimed to elucidate the novel molecular function of Withaferin A on HCC cells and its effect on various gene expression. Our results clearly showed that Withaferin A treatment to HCC cells inhibited proliferation, migration, invasion, and anchorage-independent growth. Further, we explored the Withaferin A target genes by blotting human angiogenesis, and cytokine arrays using conditioned media of Withaferin A treated QGY-7703 cells. We found that many of Nuclear factor kappa B (NF-κB), angiogenesis and inflammation associated proteins secretion is downregulated upon Withaferin A treatment. Interestingly, all these genes expression is also negatively regulated by nuclear receptor Liver X receptor-α (LXR-α). Here, we explored a novel mechanism that Withaferin-A activated LXR-α inhibits NF-κB transcriptional activity and suppressed the proliferation, migration, invasion, and anchorage-independent growth of these HCC cells. All these data strongly confirmed that Withaferin A is a potent anticancer compound and suppresses various angiogenesis and inflammatory markers which are associated with the development and progression of HCC. This beneficial and potential therapeutic property of Withaferin A will be very useful for the treatment of HCC.

## Introduction

Hepatocellular Carcinoma is one of the menacing and most common types of primary liver cancers and it is the third most leading cause of cancer-related deaths across the globe (1, 2). Commonly known HCC causes include Hepatitis B Virus, Hepatitis C Virus, exposure to dietary and environmental toxins, and carcinogens such as Aflatoxins and aristolochic acid, also chronic and excess alcoholism. Recently, due to lifestyle modifications, lack of physical activity or exercise is leading to obesity, type 2 diabetes, cardiovascular diseases, and non-alcoholic fatty liver disease (NAFLD) associated HCC (3). HCC has a direct link with excess intake of high calorie diet, dyslipidemia, insulin resistance, endoplasmic reticulum stress, oxidative stress, and adiposity (4). There are various signaling pathways associated with the initiation, development, and progression of hepatocellular carcinoma (5). Some of these signaling pathways are involved in proliferation, invasion, migration, anchorage-independent growth, and resistance to apoptotic stimuli (6). Targeting these pathways with suitable and specific drugs to treat HCC is the urgent need of the hour.

Angiogenesis is one of the important hallmarks of all types of cancer and is also involved in growth, development, and metastasis of HCC (7). There are many angiogenic factors involved in this HCC associated tumor angiogenesis (8). Along with these angiogenic factors many inflammatory cytokines are also known to play a major role in this disease progression (9). It is also known that many natural compounds have exhibited their inhibitory effect on the secretion of angiogenic factors and inflammatory cytokines in various types of cancers including HCC (10, 11).

Withaferin A, a natural steroidal lactone and dietary phytochemical from Indian medicinal plant Ashwagandha (*Withania Somnifera*) are very well studied for its antiangiogenic potential and anti-inflammatory properties (12). Withaferin A inhibits NF-κB, Specificity protein 1 (Sp1) transcription factors, and downregulates Vascular Endothelial Growth Factor (VEGF) gene expression (13, 14). It also acts as a ligand for nuclear receptor LXR-α and activates and regulates LXR-α mediated metabolic functions (15, 16). A recent study showed the leptin sensitizing property of Withaferin-A with strong antidiabetic properties on diet induced obesity mice (17). All these studies have demonstrated the anti-metabolic syndrome effect of Withaferin A (18, 19). However, the exact molecular mechanism behind its role in the inhibition of important hallmarks of hepatocellular carcinoma is not well established and is yet to be explored.

To explore and elucidate the molecular mechanism of action of Withaferin A on HCC cells, we examined the effect of Withaferin A on proliferation, anchorage-independent growth ability, migration, invasion using HCC cells. Here, we established a very strong link between angiogenic factors and inflammatory cytokines secretion and their role in controlling cancer hallmarks upon Withaferin A treatment. We found that Withaferin A modulates the secretion of angiogenic factors and inflammatory cytokines and also inhibits proliferation, migration, invasion, and anchorage-independent growth of these cells through the activation of LXR-α and LXR-α mediated suppression of NF-κB transcription factor. Based on all these beneficial effects along with the multifaceted function of this wonder compound (19), it can also be used as a therapeutic drug in the treatment of hepatocellular carcinoma.

## Materials and Methods

### Cell Culture

HepG2 cells, Hep3B cells, Huh-7 cells, QGY-7703 cells, which are very well studied human hepatoma and hepatocellular carcinoma cell lines are used in this study. HepG2 and Hep3B were obtained from the American Type Culture Collection, Manassas, VA, USA. Huh-7 and QGY-7703 cells were a kind gift from Dr. Devanand Sarkar, Virginia Commonwealth University, Richmond, VA, USA. HepG2 cells, Huh-7 cells, QGY-7703 cells were grown in Dulbecco’s modified Eagle’s medium (DMEM) with 10% fetal calf serum supplemented with 10% FBS and 1% penicillin/streptomycin, and Hep3B cells were grown in MEM alpha with 10% FBS, 5% Sodium Pyruvate, 5% Non-essential amino acids, and 1% penicillin/streptomycin at 37°C in a humidified atmosphere containing 5% CO_2_ and 18% O_2_. When cells reached 80–90% confluence of growth, they were trypsinized and seeded in different culture plates or flasks based on our experimental needs.

### Proliferation Assay

Cell proliferation was evaluated by Water Soluble Tetrazolium-1 (WST-1) Cell Proliferation Assay System (Roche Diagnostics, Rotkreuz, Switzerland). HCC cells were seeded in 96-well plates at 5 × 10^3^ cells per well and treated with Withaferin A (5 µM) at 37°C under 5% CO_2_ for 24 h. At the end of the 24 h period, 10 µl premixed WST-1 reagent was added to each well, and the plates were incubated further for 2 h at 37°C under 5% CO_2_. Thereafter, absorbance was measured at 450 nm using a Turner-Biosystems microplate reader.

### Colony Formation Assay

Colony formation assay was carried out using Huh-7 and QGY-7703 cells. The cells were seeded in 6 cm dishes at a density of 500 cells per plate and treated with Withaferin A (5 µM) and cultured for about 14–16 days until the colonies were visible. The cells were fixed in formaldehyde for 20 mins and washed with running tap water and stained with 10% Giemsa (Sigma-Aldrich, St. Louis, MO, USA). After rinsing and washing with running tap water, the plates were air dried, visualized under the microscope, and photographed. The images were analyzed using NIH ImageJ software and colonies counted and numbers showed in the bar graph.

### Wound Healing Assay

Wound healing assay was carried out using Huh-7 and QGY-7703 cells (2 × 10^5^ cells/3 ml). The cells were seeded in a six-well plate and incubated at 37°C until cells were 90% confluent. A scratch was made using a 100–200 ml pipette tip, followed by washing with PBS to remove cell debris, and then treated with 5 μM Withaferin A in a complete medium. After 24 h of incubation, the cells were observed under a light microscope and randomly chosen fields were photographed at 20× objective. The percentage of Huh-7 and QGY-7703 cells migrated into the scratched area was calculated using ImageJ software.

### Transwell Invasion Assay

Transwell Invasion assay was conducted using BD BioCoat Matrigel Invasion Chamber (BD Biosciences, Franklin Lakes, NJ, USA) as suggested in the manufacturer’s instructions. Pre warmed serum free media was added to the bottom side of the transwell as well as the upper chamber above the matrigel for 2 h at room temperature for rehydration. Huh-7 and QGY-7703 cells (5 × 10^4^ cells) were seeded in the upper chamber in serum free medium (with or without 5µM Withaferin A) while the wells of the lower chamber were filled with complete medium (5% FBS). After 22 h of incubation at 37°C and 5% CO_2,_ the cells on the upper surface of the transwell filters were removed by gentle wiping with a cotton swab and the cells attached on the lower surface of the filters were fixed and stained with Diff-Quick stain (IMEB Inc., San Marcos, CA, USA). After staining the invaded cells on the transwell filter were photographed using a microscope and invasion was determined by counting the cells using ImageJ software (6).

### Soft Agar Colony Formation Assay

Anchorage-independent growth ability of HCC cells was measured by conducting soft agar colony formation assay using highly aggressive QGY-7703 cells. These cells were pretreated with vehicle control and Withaferin A for 4 h and cells were trypsinized, counted, and seeded at 10^5^ cells/plate in 6 cm dishes with culture media containing 0.4% noble agar (Sigma-Aldrich, St. Louis, MO, USA) over a 0.8% agar base layer at 37°C with 5% CO_2_ for 15 days. The colonies formed were counted manually under the microscope and photographed.

### Human Angiogenesis and Cytokine Arrays

Human Angiogenesis and Cytokine Arrays were carried out to measure the secreted angiogenic and cytokine markers. The QGY-7703 cells were cultured up to 70% confluence and Withaferin A was treated for 24 h and the media was changed to serum free media for further 24 h. Supernatants of cells cultured in serum free media (conditioned media) were collected, centrifuged, cell debris was separated, and the only supernatant was used to check the expression and secretion of angiogenesis associated growth factors, cytokines, and other related molecules using commercially available human angiogenesis antibody array and Human Cytokine Array kit following the manufacturer’s instructions sheets (R&D Systems, Minneapolis, MN, USA).

### Quantitative Real-Time PCR

Total RNA was extracted from HepG2 cells treated with or without Withaferin A using TRIzol reagent (Thermos Scientific, Waltham, MA, USA). The experimental procedure was followed as described previously (6) and the primer sequences for the selected and validated LXR-α target genes are given in **Table 1**.

**Table 1:** Primer sequences for the selected LXR-α target genes were used for validation after Withaferin A treatment to HepG2 cells.

SL	Gene	Primer sequence
1	hABCA1	5′-TTCCCGCATTATCTGGAAAGC-3′ (Forward primer) 5′-CAAGGTCCATTTCTTGGCTGT-3′ (Reverse primer)
2	hABCG1	5′-ATTCAGGGACCTTTCCTATTCGG-3′ (Forward primer) 5′-CTCACCACTATTGAACTTCCCG-3′ (Reverse primer)
3	hApoE	5′-GTTGCTGGTCACATTCCTGG-3′ (Forward primer) 5′-GCAGGTAATCCCAAAAGCGAC-3′ (Reverse primer)

### Statistical Analysis

All the data are presented as means ± SEM (n = 3). Statistical significance was analyzed using a two-tailed unpaired Student’s t-test. GraphPad Prism software (version 6) was used for all statistical analyses and p values <0.05 were considered significant.

## Results

### Withaferin A Inhibits Proliferation, Migration, and Invasion of HCC Cells

**Figure 1:**
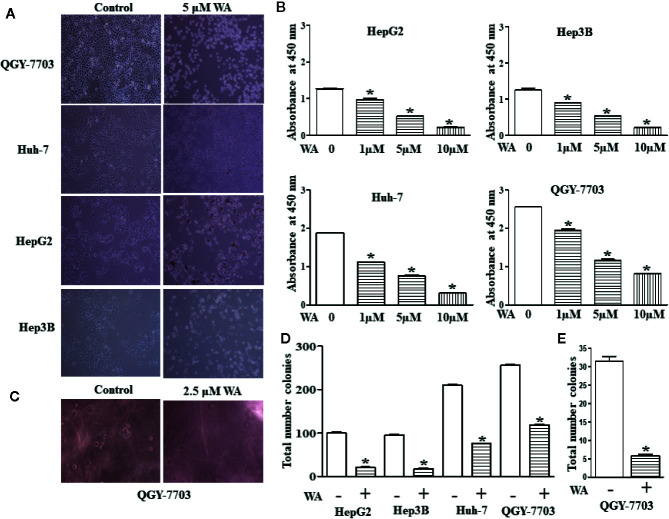
Withaferin A inhibits proliferation of HCC cells. Cell death was induced in HCC cells by Withaferin A Cells (HepG2, Huh7, Hep3B, and QGY-7703 cells) were treated with/without Withaferin A (5 µM) for 24 h and then observed under inverted microscope (n = 3) **(A)**. Withaferin A suppressed the proliferation of HCC cells, absorbance was measured at 48 h (n = 3) **(B)**. Withaferin A (2.5 µM) inhibited the anchorage-independent growth of QGY-7703 cells (n = 3) **(C, E)** and colony formation ability (n = 3) **(D)**.

**Figure 2:**
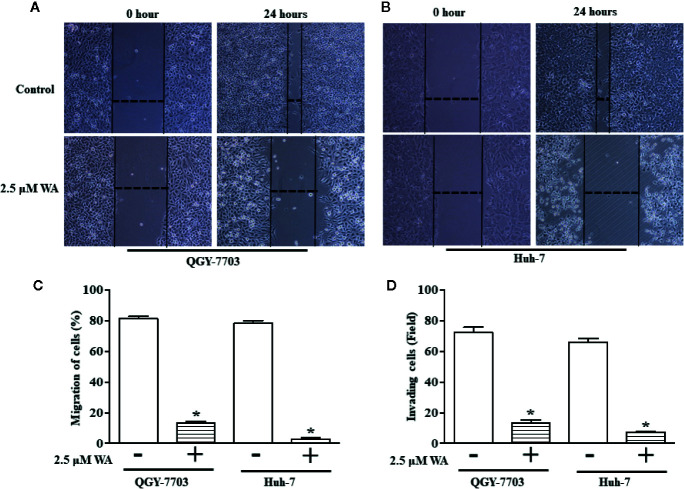
Withaferin A inhibits migration and invasion of QGY-7703 and Huh-7 cells. Cells were treated with Withaferin A (2.5 µM) for 24 h and pictures were taken before and after the treatment and the migration distance was measured using ImageJ software and percentage inhibition was measured (n = 3) **(A–C)** and transwell invasion was measured by staining and counting the number of invaded QGY-7703 and Huh-7 cells (n = 3) **(D)**. *p value is less than 0.05.

In this study, we explored the therapeutic potential of Withaferin A on proliferation, migration, and invasion of HCC cells. HCC cells (Hep3B, HepG2, Huh-7, and QGY-7703) were treated with various doses (1, 5, and 10 µM) of Withaferin A for 24 h. The results of the WST-1 cell proliferation assay conducted at the end of the treatment period, showed that Withaferin A significantly inhibited the proliferation of HCC cells (**Figure 1A**) and the images were photographed under the microscope after the treatment of 5 µM Withaferin A to these cells (**Figure 1B**). Further, we validated the effect of Withaferin A on the colony formation ability of these cells and the results showed that more than 50% inhibition of colony formation was observed in Withaferin A treated cells compared to control cells. Colony formation assay (**Figure 1D**) and Soft agar colony formation assay (**Figures 1C, E**). Next, we determined the effects of Withaferin A (2.5 µM) on migration and invasion of QGY-7703 and Huh-7 cells by employing scratch wound-healing assay and transwell invasion assay. As shown, both the assays demonstrated that Withaferin A attenuated the migration (**Figures 2A–C**) and invasion (**Figure 2D**) of QGY-7703 and Huh-7 cells.

### Withaferin A Activates LXR-α and Inhibits NF-κB Signaling in QGY-7703 Cells

**Figure 3:**
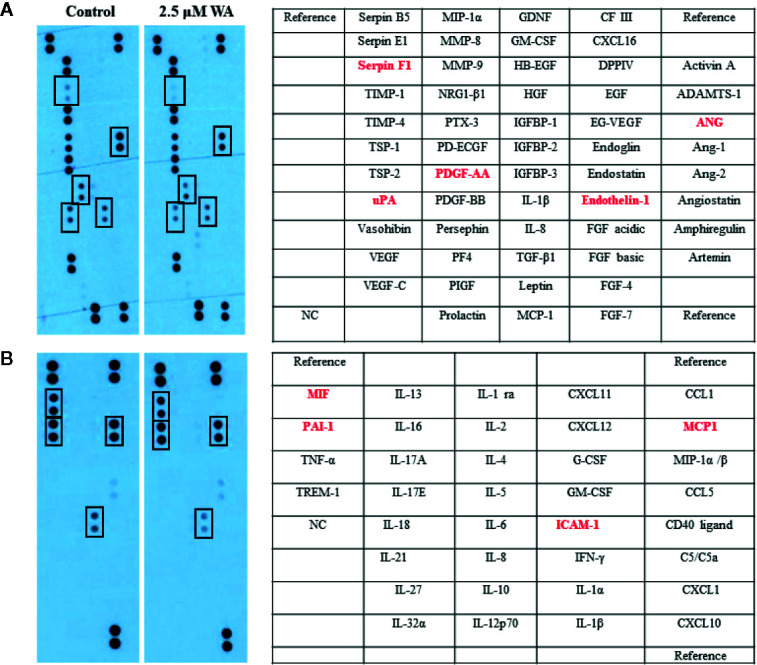
Withaferin A inhibited the secretion of angiogenesis factors and cytokines production in HCC cells. QGY-7703 cells were grown in serum free media for 24 h along with or without Withaferin A (2.5 µM). The conditioned media was used to detect the secretary angiogenic factors and cytokines (n = 3) **(A, B)**.

Here, we evaluated the effect of Withaferin A on the secretion of various angiogenesis markers and cytokines by QGY-7703 cells. Recently, few studies have shown that Withaferin A has LXR-α agonist property and it acts as a specific ligand for LXR-α (16–19). However, the significance of this property of Withaferin A and its molecular action is not studied in cancer cells. Withaferin A is also known for its anti-inflammatory properties *via* inhibiting the NF-κB transcription factor (20). LXR-α, a nuclear receptor family member is known to play a pivotal role in the various biological process which includes inflammation, cholesterol homeostasis, lipogenesis, cellular reprogramming, and decisions (16). Therefore, we focused our study on LXR-α/NF-κB signaling pathway, and the data supported our hypothesis. Withaferin A (2.5 µM) treatment decreased the secretion of various angiogenesis-related markers, growth factors, and cytokines (Serpin F1(PEDF), uPA, PDGF-AA, Angiogenin, Endothelin-1, Macrophage migration inhibitory factor (MIF), PAI-1, MCP1, ICAM-1 in QGY-7703 cells (**Figures 3A, B**). These factors are very well known for their pivotal role in proliferation, migration, invasion, angiogenesis, inflammation, and metastasis (21–23). It is also a known fact that NF-κB is a master regulator of various inflammatory signaling pathways (24). All these factors are directly or indirectly regulated by both NF-κB and LXR-α (25, 26). LXR-α is a negative regulator of NF-κB signaling and in this study activation of LXR-α by Withaferin A may downregulate the secretion of all these molecules *via* suppressing NF-κB activity.

### Withaferin A Induces LXR-α Target Genes in HepG2 Cells

**Figure 4:**
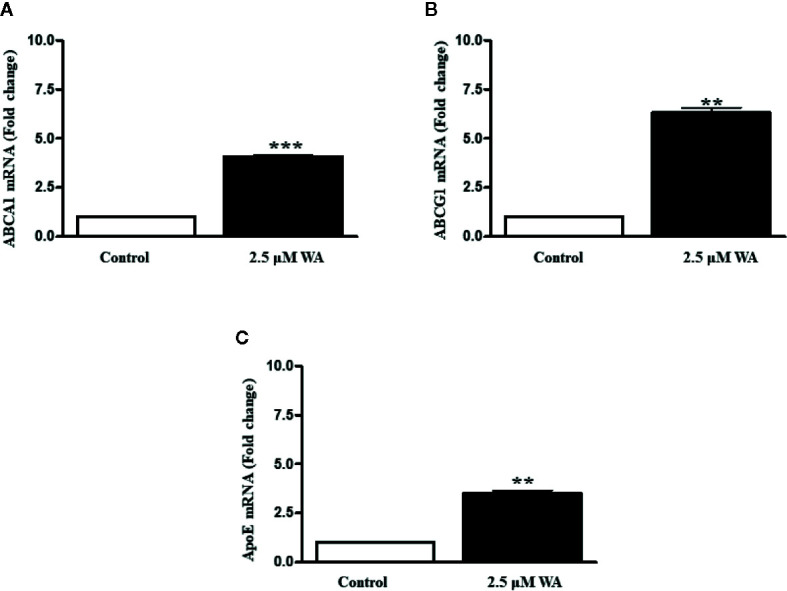
Withaferin A activates LXR-α and induces its target genes. HepG2 cells were grown in regular media for 4 h along with or without Withaferin A (2.5 µM). The gene expression of ABCA1, ABCG1, and ApoE were measured (n = 3) **(A–C)**. **p value is less than 0.005 and ***p value is less than 0.001.

Further, to confirm the agonistic role of Withaferin A we thought of validating some of the LXR-α target genes in HCC cells. Therefore, we treated HepG2 cells with Withaferin A (2.5 µM) for 4 h and isolated total RNA from these cells, and measured the expression of ATP-binding cassette sub-family A member 1 (ABCA1), ATP-binding cassette sub-family G member 1(ABCG1), and Apolipoprotein E(ApoE). These three genes are commonly known LXR-α target genes and were found to be significantly increased in Withaferin A treated cells in comparison with vehicle controls cells (**Figure 4**).

## Discussion

Natural compounds are gaining increasing popularity in recent years as pharmaceutical drugs due to their pleiotropic effects and multifaceted beneficial properties (17, 27, 28). Dietary natural compounds are even more popular, and they lack toxic side effects, and also, they can be consumed very easily as a tonic or oral pill (29). In this study, we demonstrated the novel function of Withaferin A, a natural compound from the roots and leaves of Indian winter cherry, on the growth and aggressive behavior of HCC cells and their reprogramming *via* LXR-α activation (16, 17). Many previous studies have documented the medicinal properties of this miracle compound including anti-cancer activity (12, 14, 16). Withaferin A induces apoptosis by generating reactive oxygen species and down-regulating B-cell lymphoma 2 (Bcl-2) protein in human melanoma cells and breast cancer cells (30). Withaferin A suppressed human endothelial cells proliferation and tube forming ability (12, 14). It also upregulates the Nuclear factor erythroid 2-related factor 2 (Nrf2) transcription factor and protects from Acetaminophen-induced hepatotoxicity and liver injury (31). In this work, we showed that Withaferin A significantly inhibited hepatic cancer cell proliferation, migration, invasion, colony formation, and induced apoptosis as well as suppressed the secretion of angiogenic markers and inflammatory cytokines suggesting its beneficial effects on HCC cells.

Here, we tried to explore the molecular mechanism behind the inhibitory action of Withaferin-A on proliferation, migration, invasion, and anchorage-independent growth of HCC cells. The possible action of Withaferin A and its mechanism of inhibition may be by suppressing the NF-κB pathway. Inhibition of NF-κB by Withaferin A also suppressed the anchorage-independent growth, invasion, and migration (**Figures 1C, E** and **Figures 2A–D**).

**Figure 5:**
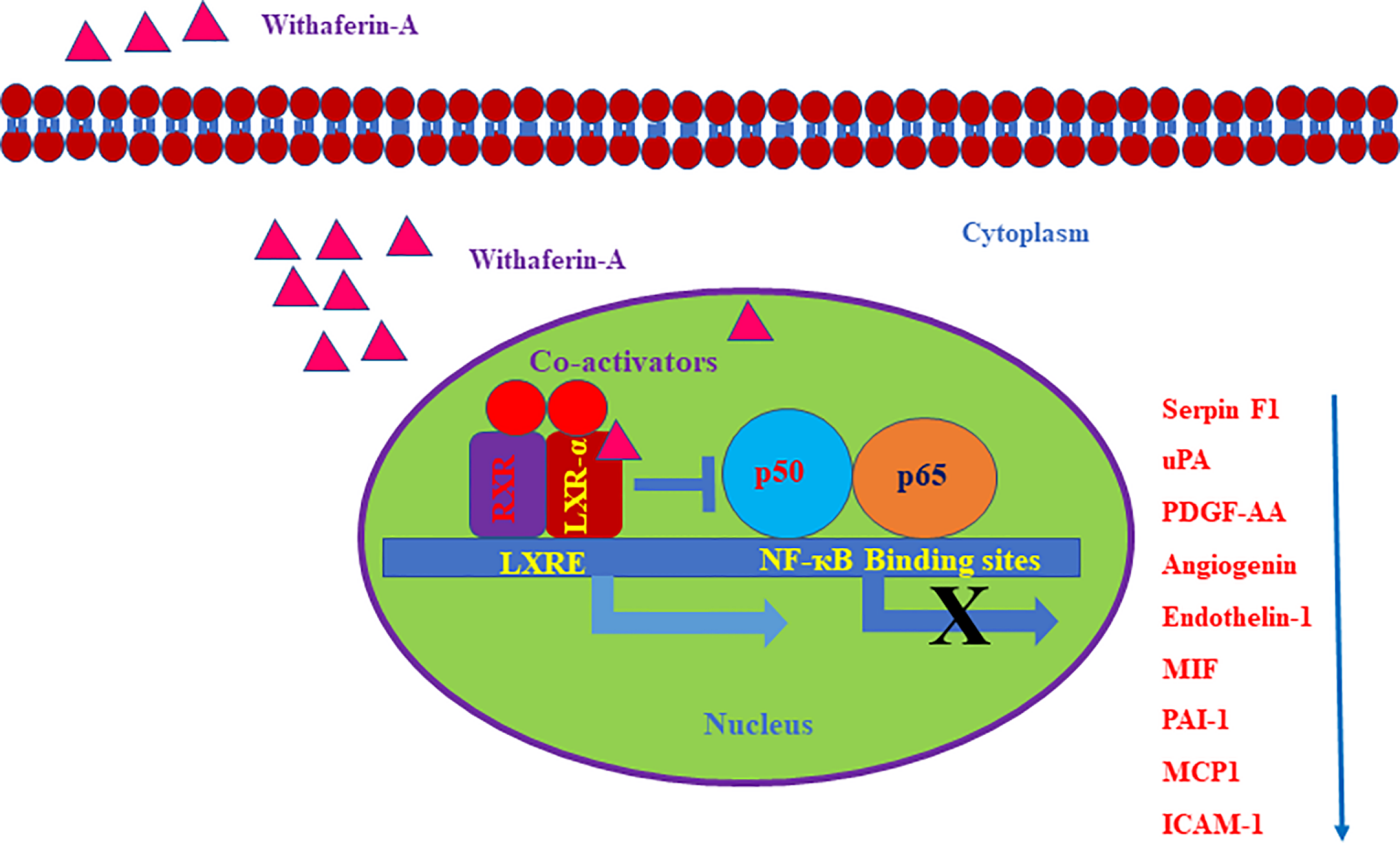
Schematic representation of withaferin A mediated regulation of LXR-α and NF-κB signaling in HCC. The negative regulatory role of LXR-α on NF-κB activation and Withaferin A mediated expression of LXR-α target genes.

Based on our angiogenesis and cytokine arrays data, we found that many LXR-α and NF-κB target genes secretion were downregulated. Some of the important angiogenic factors which are downregulated include Angiogenin, Serpin F1, or pigment epithelium-derived factor (PEDF), Platelet-Derived Growth Factor-AA (PDGF-AA), Endothelin-1, and Urokinase-type plasminogen activator (uPA). All these factors are known to be directly regulated by NF-κB signaling (32). LXR-α was known to inhibit the expression of Endothelin-1 and also suppresses the PDGF-induced proliferation and regulates uPA gene expression (33–35). Also, a previously reported study on gene regulation by LXR agonist treatment shows that synthetic LXR-ligands downregulates Angiogenin expression in the liver (36). Our Bioinformatics analysis using Champion ChiP Transcription Factor Search Portal of SA Biosciences database known as DECODE (DECipherment of DNA Elements) revealed that human Endothelin-1, Angiogenin, uPA, PDGFA, CCL2 (MCP-1), ICAM1(CD54), Serpin E1(PAI-1), and macrophage migration inhibitory factor (MIF) gene promoter regions have NF-κB binding sites. Many LXR-α agonists were also known for their effective inhibitory action on MCP-1, ICAM1, PAI-1, and other inflammatory markers (37). To confirm our experimental evidence, we further validated some of the LXR-α target genes and found that these target genes were significantly increased after Withaferin A treatment. Based on this strong and convincing evidence from our data and already known information from few reports on LXR-α and its negative regulatory role on NF-κB signaling (26), we are proposing the possible novel mechanistic model that Withaferin A may negatively regulate NF-κB transcription factor *via* activating LXR-α (**Figure 5**). There are many elegant studies, which support our evidence-based claim and have shown that activation of LXR-α results in suppression of HCC growth and development (38, 39).

In conclusion, Withaferin A inhibited the secretion of various angiogenic factors and cytokines secreted from human HCC cells. In this study, we also showed that Withaferin A inhibited principal hallmarks of HCC cells, such as proliferation, invasion, migration, and anchorage independent growth. Our findings provide additional evidence that this well-known dietary phytochemical has a novel function and it can be used as a promising anticancer compound in the treatment of highly aggressive HCC.

## Data Availability Statement

The original contributions presented in the study are included in the article/supplementary material. Further inquiries can be directed to the corresponding author.

## Author Contributions

VS and NS: Contributed experimentally to this article. DK and FM: Contributed intellectually to this article. PS: Contributed experimentally and intellectually and wrote this article. All authors contributed to the article and approved the submitted version.

## Acknowledgments

This work was supported by Ramalingaswami Re-entry fellowship, Department of Biotechnology (DBT), Govt. of India to PS and stipend support to VS.

## Conflict of Interest

The authors declare that the research was conducted in the absence of any commercial or financial relationships that could be construed as a potential conflict of interest.

## References

[1] Forner A Reig M Bruix J Hepatocellular carcinoma. Lancet (2018) 391(10127):1301– 1314. 10.1016/S0140-6736(18)30010-2 29307467

[2] Parkin DM Bray F Ferlay J Pisani P Global cancer statistics, 2002. CA Cancer J Clin (2005) 55(2):74–108. 10.3322/canjclin.55.2.74 15761078

[3] Sanyal AJ Yoon SK Lencioni R The etiology of hepatocellular carcinoma and consequences for treatment. Oncologist (2010) 15(Suppl 4):14–22. 10.1634/theoncologist.2010-S4-14 21115577

[4] Asgharpour A Cazanave SC Pacana T Seneshaw M Vincent R Banini BA A diet-induced animal model of non-alcoholic fatty liver disease and hepatocellular cancer. J Hepatol (2016) 65(3):579–88. 10.1016/j.jhep.2016.05.005 PMC5012902 27261415

[5] Santhekadur PK Kumar DP Sanyal AJ Preclinical models of non-alcoholic fatty liver disease. J Hepatol (2018) 68(2):230–7. 10.1016/j.jhep.2017.10.031 PMC5775040 29128391

[6] Kumar DP Santhekadur PK Seneshaw M Mirshahi F Tuculescu CU Sanyal AJ A Regulatory Role of Apoptosis Antagonizing Transcription Factor in the Pathogenesis of Nonalcoholic Fatty Liver Disease and Hepatocellular Carcinoma. Hepatology (2019) 69(4):1520–34. 10.1002/hep.30346 PMC6440548 30394550

[7] Santhekadur PK Das SK Gredler R Chen D Srivastava J Robertson C Multifunction protein staphylococcal nuclease domain containing 1 (SND1) promotes tumor angiogenesis in human hepatocellular carcinoma through novel pathway that involves nuclear factor κB and miR-221. J Biol Chem (2012) 287(17):13952–8. 10.1074/jbc.M111.321646 PMC3340184 22396537

[8] Zhu AX Duda DG Sahani DV Jain RK HCC and angiogenesis: possible targets and future directions. Nat Rev Clin Oncol (2011) 8(5):292–301. 10.1038/nrclinonc.2011.30 21386818 PMC3266719

[9] Park EJ Lee JH Yu GY He G Ali SR Holzer RG Dietary and genetic obesity promote liver inflammation and tumorigenesis by enhancing IL-6 and TNF-expression. Cell (2010) 140(2):197–208. 10.1016/j.cell.2009.12.052 20141834 PMC2836922

[10] Zhou Y Li Y Zhou T Zheng J Li S Li HB Dietary natural products for prevention and treatment of liver cancer. Nutrients (2016) 8(3):156. 10.3390/nu8030156 26978396 PMC4808884

[11] Cao W Hu C Wu L Xu L Jiang W Rosmarinic acid inhibits inflammation and angiogenesis of hepatocellular carcinoma by suppression of NF-κB signaling in H22 tumor-bearing mice. J Pharmacol Sci (2016) 132(2):131–7. 10.1016/j.jphs.2016.09.003 27707649

[12] Mohan R Hammers HJ Bargagna-Mohan P Zhan XH Herbstritt CJ Ruiz A Withaferin A is a potent inhibitor of angiogenesis. Angiogenesis (2004) 7(2):115–22. 10.1007/s10456-004-1026-3 15516832

[13] Bargagna-Mohan P Ravindranath PP Mohan R Small molecule anti-angiogenic probes of the ubiquitin proteasome pathway: potential application to choroidal neovascularization. Invest Ophthalmol Vis Sci (2006) 47(9):4138–45. 10.1167/iovs.05-1452 PMC3229038 16936134

[14] Santhekadur PK Shilpa P Salimath BP Withaferin A suppresses the expression of vascular endothelial growth factor in Ehrlich ascites tumor cells via Sp1 transcription factor. Curr Trends Biotechnol Pharm (2009) 3(2):138–48.

[15] Dave VP Kaul D Sharma Y Bhattacharya R Functional genomics of blood cellular LXR-alpha gene in human coronary heart disease. J Mol Cell Cardiol (2009) 46(4):536–44. 10.1016/j.yjmcc.2008.12.020 19211025

[16] Mehrotra A Kaul D Joshi K LXR-α selectively reprograms cancer cells to enter into apoptosis. Mol Cell Biochem (2011) 349(1-2):41–55. 10.1007/s11010-010-0659-3 21125317

[17] Lee J Liu J Feng X Hernández MAS Mucka P Ibi D Withaferin A is a leptin sensitizer with strong antidiabetic properties in mice. Nat Med (2016) 22(9):1023–32. 10.1038/nm.4145 PMC5892415 27479085

[18] Pfluger PT Tschöp MH Obesity: will withaferin win the war? Nat Med (2016) 22(9):970–1. 10.1038/nm.4182 27603129

[19] Santhekadur PK Is Withaferin A, a magic bullet for metabolic syndrome? BioMed Pharmacother (2017) 92:1135–7. 10.1016/j.biopha.2017.04.002 28413154

[20] Chung SS Wu Y Q Okobi Q Adekoya D Atefi M Clarke O Proinflammatory Cytokines IL-6 and TNF-α Increased Telomerase Activity through NF-κB/STAT1/STAT3 Activation, and Withaferin A Inhibited the Signaling in Colorectal Cancer Cells. Mediators Inflamm (2017) 59584291–11. 10.1155/2017/5958429 PMC5476880 28676732

[21] Savard LD Lee E Kakkad S Popel AS Bhujwalla ZM The Angiogenic Secretome in VEGF overexpressing Breast Cancer Xenografts. Sci Rep (2016) 6:39460. 10.1038/srep39460 27995973 PMC5171865

[22] Hagman H Bendahl PO Lidfeldt J Protein array profiling of circulating angiogenesis related factors during bevacizumab containing treatment in metastatic colorectal cancer. PLoS One (2018) 13:e0209838. 10.1371/journal.pone.0209838 30592740 PMC6310295

[23] Zhu J Yong W Wu X Yu Y JLv J Liu C Anti-inflammatory effect of resveratrol on TNF-α-induced MCP-1 expression in adipocytes. Biochem Biophys Res Commun (2008) 369:471–7. 10.1016/j.bbrc.2008.02.034 18291098

[24] Ghosh S Hayden MS New regulators of NF-kappaB in inflammation, Nat. Rev Immunol (2008) 8:837–48. 10.1038/nri2423 18927578

[25] Schulman IG Liver X receptors link lipid metabolism and inflammation. FEBS Lett (2017) 591:2978–91. 10.1002/1873-3468.12702 PMC5638683 28555747

[26] Wu S Yin R Ernest R Li Y Zhelyabovska O Luo J Liver X receptors are negative regulators of cardiac hypertrophy via suppressing NF-κB signaling. Cardiovasc Res (2009) 84:119–26. 10.1093/cvr/cvp180 PMC2741346 19487338

[27] Pan SY Zhou SF Gao S Yu ZL Zhang SF Tang MK New Perspectives on How to Discover Drugs from Herbal Medicines: CAM’s Outstanding Contribution to Modern Therapeutics. Evid Based Complement Alternat Med (2013) 627375:1–25. 10.1155/2013/627375 PMC3619623 23634172

[28] Chakraborty P Herbal genomics as tools for dissecting new metabolic pathways of unexplored medicinal plants and drug discovery. Biochim Open (2018) 6:9–16. 10.1016/j.biopen.2017.12.003 29892557 PMC5991880

[29] Osowski A Pietrzak M Wieczorek Z Wieczorek J Natural compounds in the human diet and their ability to bind mutagens prevents DNA-mutagen intercalation. J Toxicol Environ Health A (2010) 73:1141–9. 10.1080/15287394.2010.491044 20706936

[30] Mayola E Gallerne C Esposti DD Martel C Pervaiz S Larue L Withaferin A induces apoptosis in human melanoma cells through generation of reactive oxygen species and down-regulation of Bcl-2. Apoptosis (2011) 16:1014–27. 10.1007/s10495-011-0625-x 21710254

[31] Jadeja RN Urrunaga NH Dash S Khurana S Saxena NK Withaferin-A Reduces Acetaminophen-Induced Liver Injury in Mice. Biochem Pharmacol (2015) 97:122–32. 10.1016/j.bcp.2015.07.024 PMC5909697 26212553

[32] Wang YH Dong YY Wang WM Xie XY Wang ZM Chen RX Vascular endothelial cells facilitated HCC invasion and metastasis through the Akt and NF-κB pathways induced by paracrine cytokines. J Exp Clin Cancer Res (2013) 32:51. 10.1186/1756-9966-32-51 23941552 PMC3751285

[33] Gao M Zeng Y Guan Y Hu Z Zhong D Shen X Activation of liver X receptor attenuates endothelin-1 expression in vascular endothelial cells. Int J Biochem Cell Biol (2012) 44:2299–307. 10.1016/j.biocel.2012.09.010 23018104

[34] Delvecchio CJ Bilan P Radford K Stephen J Trigatti BL Cox G Liver X receptor stimulates cholesterol efflux and inhibits expression of proinflammatory mediators in human airway smooth muscle cells. Mol Endocrinol (2007) 21:1324–34. 10.1210/me.2007-0017 17405904

[35] Tamura K Chen YE Horiuchi M Chen Q Daviet L Yang Z LXR alpha functions as a cAMP-responsive transcriptional regulator of gene expression. Proc Natl Acad Sci U S A (2000) 97:8513–8. 10.1073/pnas.100519097 PMC26979 10890879

[36] Inagaki T Moschetta A Lee YK Peng L Zhao G Downes M Regulation of antibacterial defense in the small intestine by the nuclear bile acid receptor. Proc Natl Acad Sci U S A (2006) 103:3920–5. 10.1073/pnas.0509592103 PMC1450165 16473946

[37] Huang YY Gusdon AM Qu S Nonalcoholic fatty liver disease: molecular pathways and therapeutic strategies, Lipids. Health Dis (2013) 12:171. 10.1186/1476-511X-12-171 PMC3827997 24209497

[38] Xiong T Li Z Huang X Lu K Xie W Zhou Z TO901317 inhibits the development of hepatocellular carcinoma by LXRα/Glut1 decreasing glycometabolism. Am J Physiol Gastrointest Liver Physiol (2019) 316:G598–607. 10.1152/ajpgi.00061.2018 30817182

[39] Hu C Liu D Zhang Y Lou G Huang G Chen B LXRα-mediated downregulation of FOXM1 suppresses the proliferation of hepatocellular carcinoma cells. Oncogene (2014) 33:2888–97. 10.1038/onc.2013.250 23812424

